# Pneumatosis intestinalis revisited: associated imaging findings, clinical correlation, management, risk stratification and future perspectives

**DOI:** 10.1007/s10140-026-02472-4

**Published:** 2026-05-02

**Authors:** Kathy Truong, Waseem Wahood, Cibele Luna, Austin Dosch, Yamilé Blain

**Affiliations:** 1 University of Miami Miller School of Medicine, 1600 NW 10th Avenue, Suite 1149, Miami, FL 33136 USA; 2 Department of Radiology, University of Miami Health System, 1150 NW 14th St, Suite 511, Miami, FL 33136 USA; 3 Department of Colon & Rectal Surgery, DeWitt Daughtry Family Department of Surgery, University of Miami Health System, Miami, FL USA

**Keywords:** Pneumatosis intestinalis, Bowel ischemia, Portomesenteric venous gas, Pneumoperitoneum, Computed tomography (CT), Multidisciplinary management

## Abstract

**Background:**

Pneumatosis intestinalis (PI) is the imaging finding of air within the bowel wall. It constitutes a diagnostic challenge due to its broad differential and overlap in imaging appearances between benign and serious causes. A multidisciplinary approach is essential for accurate diagnosis and timely intervention, to optimize outcomes and minimize unnecessary procedures.

**Objective:**

This article aims to synthesize the literature to lay out the current state of the art in diagnosing and managing PI and discuss future perspectives. We review a series of associated imaging features including portomesenteric venous gas, vascular thrombosis, abnormal bowel wall enhancement, and bowel wall thickening or thinning. We also discuss less specific but commonly seen findings like pneumoperitoneum, ascites and fat stranding, emphasizing their context-dependent significance. We review nuances in clinical interpretation of the severity of PI, discuss the discordance in interpretation of certain clinical markers, and important considerations in specific populations. To finish, we describe ongoing efforts related to risk stratification and its importance in PI management.

**Graphical abstract:**

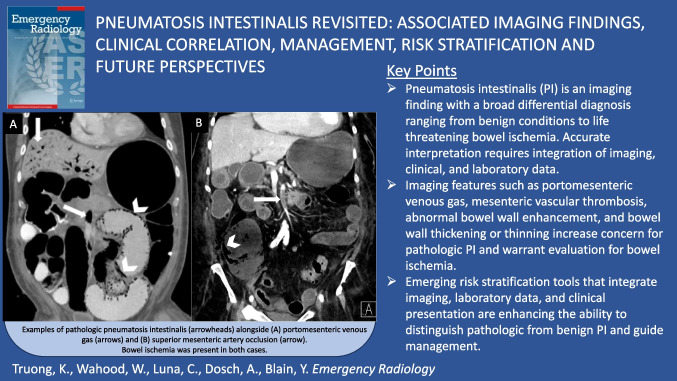

## Introduction

Pneumatosis intestinalis (PI) is the imaging finding of air within the wall of the gastrointestinal tract, beneath the serosa and mucosa. PI itself is not a disease, but rather the sign of an underlying process which may be benign or life threatening.

The incidence of PI in the general population is low [1–4]; however, its detection has been increasing due to the widespread utilization of imaging. The clinical challenge is to identify what subsets of patients may have underlying severe pathology requiring urgent intervention.

Many researchers have investigated ways to accurately select patients with pathological PI; studies with varying, sometimes opposite, results abound, and to date there remains a lack of consensus and clear guidelines. While the identification of certain critical imaging findings is a clear indication of serious illness, there are many more findings whose presence is ambiguous. Clinical correlation, often the holy grail when imaging falls short, has its own shortcomings as well. Interpretation of long relied upon clinical markers is not always straightforward and can be confounded by preexisting conditions, comorbidities and concomitant use of pharmaceutical agents.

In this review, we discuss radiologic findings, clinical signs, and laboratory markers that can be used to assess PI severity. For the purposes of this review, severity refers to the likelihood that PI represents clinically significant, pathologic ischemia, particularly transmural bowel injury with risk of necrosis, perforation, or need for urgent intervention. We weigh in on the significance of portomesenteric venous gas, and mesenteric vascular thrombosis in relation to abnormal bowel wall enhancement, and bowel wall thickening or thinning. We discuss potential mimickers such as pseudo-pneumatosis and pneumobilia. We also highlight findings like pneumoperitoneum, ascites, and fat stranding and emphasize their context-dependent significance.

We discuss clinical markers that are commonly used to evaluate the severity of patients’ conditions in the context of radiologic findings. We underline the growing incidence of PI in patients receiving chemo/immunotherapy or who are otherwise immunocompromised, and the implications for diagnosis and management.

Finally, we provide an overview of current management options, delineate ongoing efforts in risk stratification, and offer future perspectives.

## Recognizing pneumatosis intestinalis

**Fig. 1:**
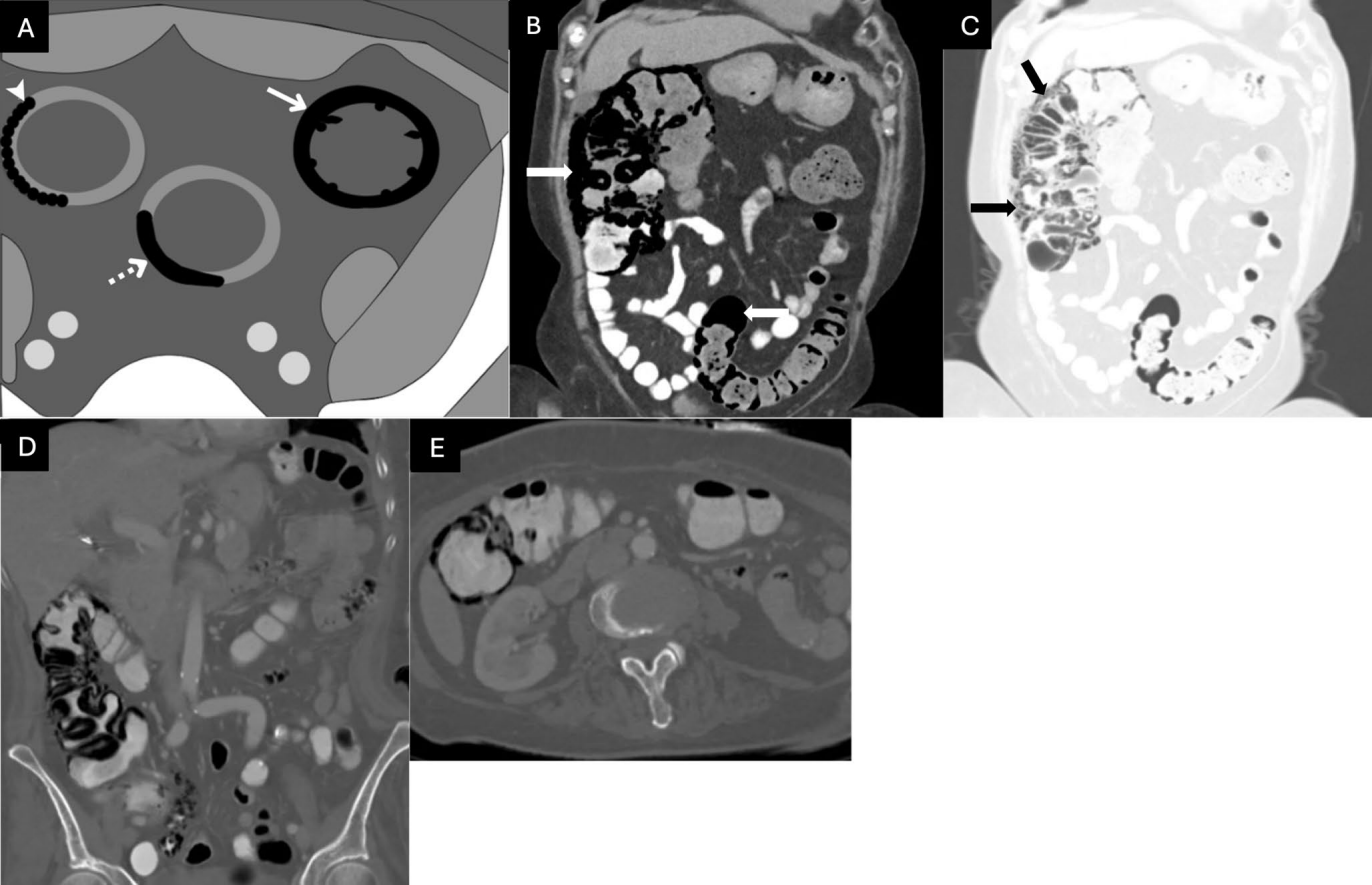
**A** Schematic representation of an axial image of the pelvis demonstrates the patterns of PI within three bowel loops: Cystic pattern, multiple thin-walled cystic spaces within the bowel wall (arrowhead). Linear pattern, crescent-shaped distribution of intramural gas (dashed arrow). Circumferential pattern, circular distribution of gas within the bowel wall (solid arrow). **B, C** 55-year-old patient with acute myeloid leukemia on long term steroids. Coronal CT images in (**B**) abdominal window and (**C**) lung window show extensive linear and cystic PI (arrows) of the cecum, ascending and proximal transverse colon. These findings were identified on routine follow-up imaging. This patient was asymptomatic and treated non-operatively. **D, E** 80-year-old-patient with history of metastatic lung cancer presented with malfunctioned peg tube. Coronal (**D**) and axial (**E**) CT images demonstrate circumferential PI (arrows) involving the distal ileum, cecum and ascending colon to the level of the hepatic flexure. This patient was treated nonoperatively with complete bowel rest, intravenous antibiotics, and monitoring with serial abdominal exams and lactate trends

The presence of air within the bowel wall can be recognized on imaging in various modalities including radiographs, ultrasound, and CT scans. Among all, CT has the highest sensitivity and specificity. PI is classified as either primary or secondary based on etiology. Primary PI, also termed pneumatosis cystoides intestinalis (PCI), is idiopathic, occurring in the absence of an underlying pathological condition, and is generally benign. In contrast, secondary PI represents a manifestation of an underlying disorder, such as intestinal ischemia, infection, inflammatory bowel disease, malignancy, chemotherapy-related injury, iatrogenic causes, or immunodeficiency [1–3]. Three patterns of PI have been recognized and described in the literature: cystic, linear, and circumferential (Fig. 1A). Cystic PI is characterized by multiple thin-walled cysts or bubbles of gas in the submucosal or subserosal spaces of the bowel wall. The cystic appearance is the most common among individuals with idiopathic or primary pneumatosis, but it can also be observed with any cause of PI (Fig. 1B). The linear pattern is characterized by curvilinear or crescent-shaped areas of gas, and the circumferential pattern is characterized by circular gas in the bowel wall. Secondary PI is more often seen with linear and/or circumferential gas shapes. In certain cases, a combination of any of these three patterns can be seen in the same patient (Fig. 1C). Due to the overlap between the different gas patterns and the underlying pathologies, appearance alone should not be used to distinguish between benign and serious causes of PI. The presence of PI should be integrated with other clinical and imaging findings to establish the diagnosis [1–4].

**Fig. 2:**
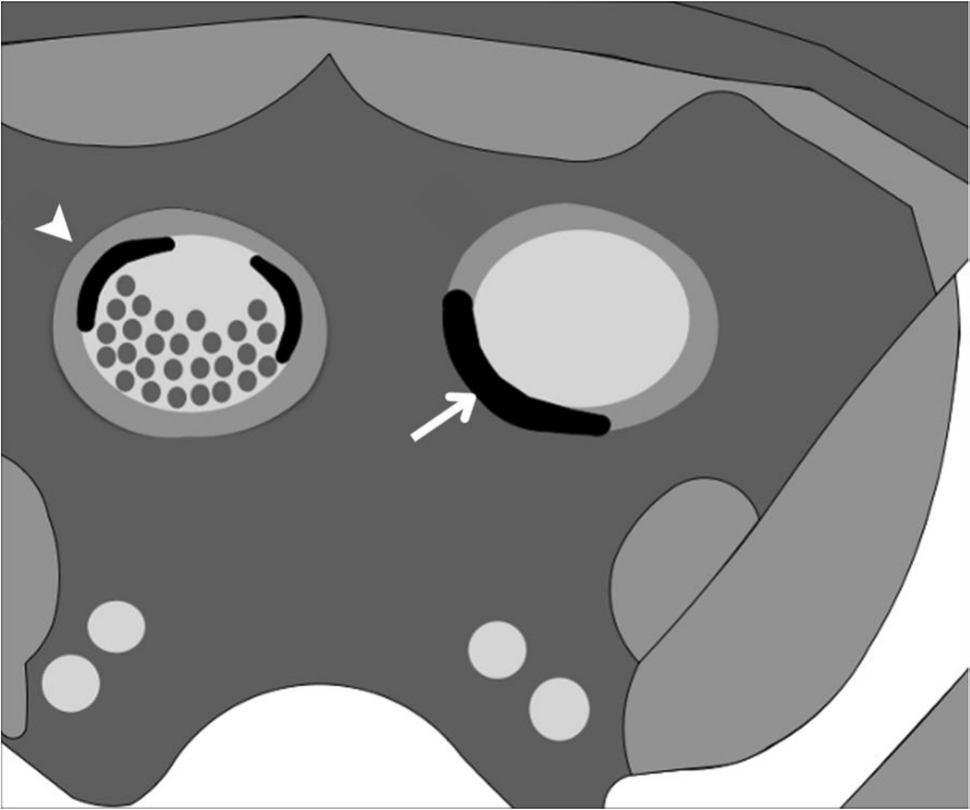
Schematic representation of an axial image of the pelvis illustrating the difference between pseudopneumatosis and true pneumatosis. In pseudo pneumatosis, the air is located between the feces and the bowel wall (arrowhead) instead of within the bowel wall as in true pneumatosis (arrow)

Pneumatosis can be mistaken for pseudo-pneumatosis, which is defined as gas trapped between the bowel wall and luminal contents or opposing mucosal folds. Gas adherence to the bowel wall can also mimic PI. It is important to be able to differentiate PI from pseudo-pneumatosis (Fig. 2). PI will appear along the bowel wall and extend beyond gas-fluid levels. PI can occur focally or throughout the small bowel or colon, whereas pseudo-pneumatosis is more frequently seen in the cecum and ascending colon due to a mixture of liquid stool and gas. PI has a relatively smooth gas column pattern, reflecting associated wall edema. On the other hand, pseudo-pneumatosis gas adjacent to the bowel wall will stop at the free gas-fluid level and will appear as irregularly punctuated gas columns due to intact mucosal folds [2, 5]. Utilizing lung windows allows for easy detection of intraluminal air to better differentiate PI from its mimics [2]. In addition to aiding differentiation from mimics, routine review of lung window settings on all abdominal CT examinations is essential, as subtle gas may be inconspicuous on soft-tissue windows and easily overlooked. Systematic evaluation of bowel gas patterns on lung windows improves detection of PI and should be incorporated into standard interpretive practice.

**Fig. 3:**
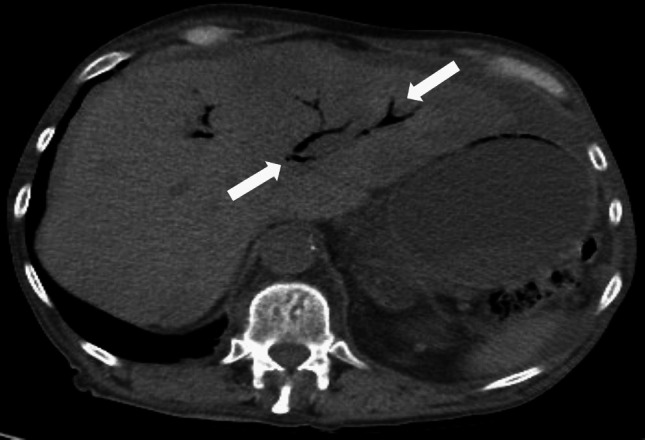
65-year-old patient with metastatic liver cancer. Pneumobilia here is secondary to a biliary stent placement (not shown). Note the centralized distribution of gas in the biliary tree—centripetal flow from bile ducts toward hilum (arrows) vs. portal venous gas in Fig. 4 where the gas is seen branching in the periphery

## Assessing the severity of pneumatosis intestinalis on imaging

PI may be associated with a variety of pathological causes, ranging from benign to life-threatening. In clinical practice, patients may present multiple comorbidities, and the distinction between innocuous PI and more serious conditions is crucial to informing treatment decisions.

Among the broad range of conditions that can be associated with PI are lung diseases such as asthma, COPD, cystic fibrosis, systemic diseases including AIDS, systemic lupus erythematosus, inflammatory bowel disease, use of certain medications (steroids, immunotherapy), trauma, recent endoscopy or bowel surgery, bowel obstruction, toxic megacolon, and ischemia [2–4].

Bowel ischemia is considered one of the most serious conditions, because if left untreated, it can quickly progress to transmural infarction, necrosis, and perforation, which can lead to systemic shock and death [6, 7]. The mortality rate of untreated bowel ischemia is high; therefore, rapid diagnosis is imperative to guide interventions and improve patient outcomes [6, 7]. In this section, we outline several common radiological findings that can be associated with PI and discuss their predilection toward benign or severe disease.

### Portomesenteric venous gas

Portomesenteric venous gas (PMVG) is the presence of gas within the portal vein, its branches, and the mesenteric veins. On imaging, PMVG is demonstrated in a peripheral tree-like branching pattern as it spreads with the flow of venous blood in the liver [8]. Portal venous gas (PVG) should not be confused with pneumobilia or air within the biliary tree. While PVG has a peripheral pattern in the liver, pneumobilia is usually centrally located in the hilum with fewer branches (Fig. 3) [9, 10].

**Fig. 4:**
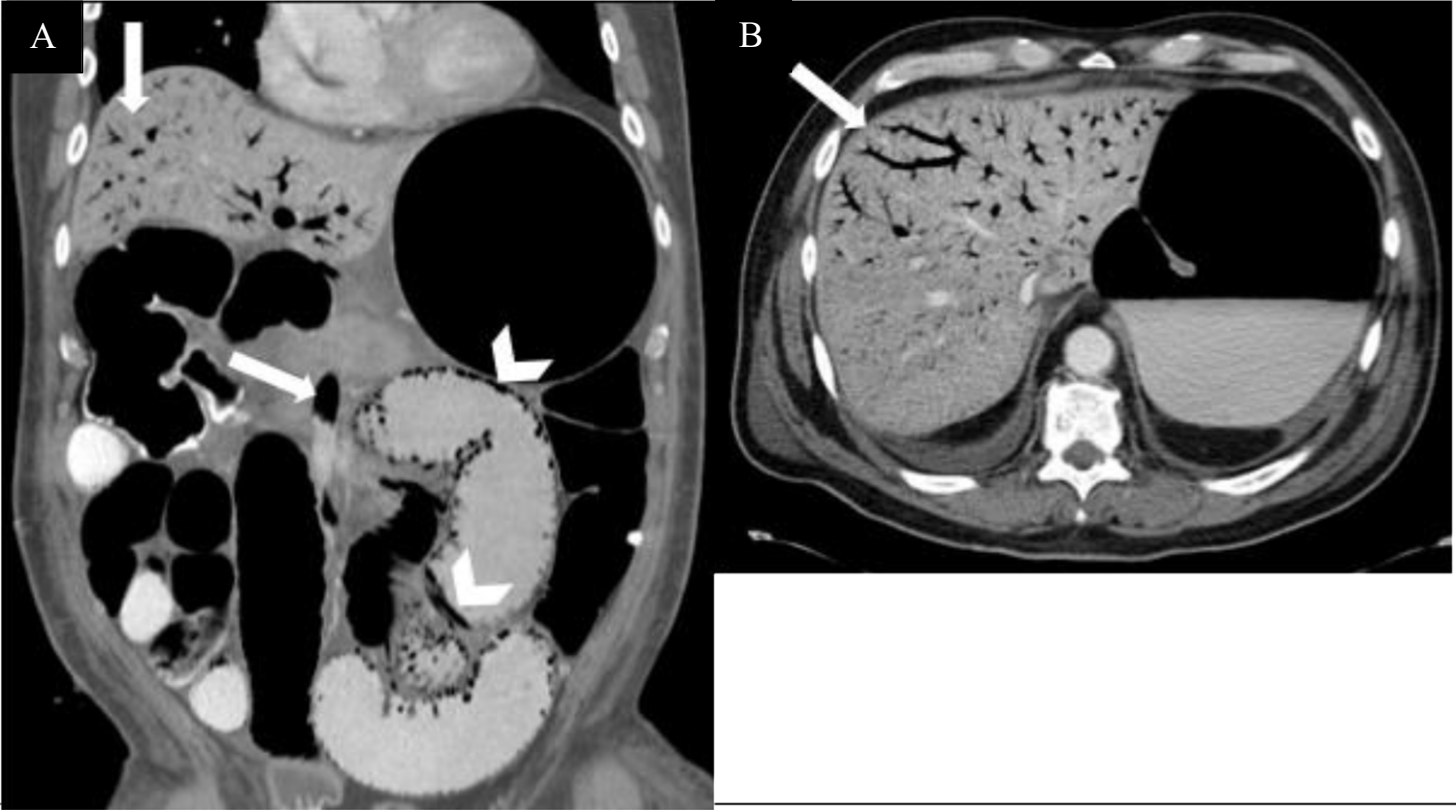
58-year-old patient status post multi-visceral transplant of liver, pancreas, and bowel with worsening abdominal pain after feeding trial. Coronal CT image **A** shows gas in the superior mesenteric and portal veins (arrows) and PI of several dilated small bowel loops (arrowheads). Axial CT image **B** shows branching gas in the liver periphery (centrifugal flow from portal vein toward periphery). Laparoscopic evaluation revealed extensive bowel ischemia and approximately 120 cm of small bowel was resected

Gas within the bowel wall in PI can migrate along the mesenteric veins which drain the intestine into the portal vein and its branches, explaining a relationship between PI and PMVG [1]. PMVG can also be seen in several other settings without PI, including after liver transplantation, percutaneous endoscopic gastrostomy tube placement, endoscopic retrograde cholangiopancreatography, and conditions like appendicitis and cholangitis [1, 8, 11, 12].

Isolated PMVG does not necessarily indicate a life-threatening condition. However, many studies that investigated the association between PI and bowel ischemia noted that the presence of PMVG in that context can signal a bad prognosis. Abboud et al. reported that PMVG and PI were related to transmural bowel necrosis in 91% of cases and carried a mortality rate of 85% [12]. In their retrospective analysis of over 100 cases, Lassandro et al. determined intestinal infarct was the underlying cause of PI and PMVG in 69.2% of their cases. Infarcted patients with PI and without PMVG had a mortality rate of 31.4%; this rate rose to 72.2% in infarcted patients with PI and PMVG [13]. Similarly, within a smaller cohort of patients with bowel infarction, Weisner et al. discovered that while PI alone was associated with a mortality rate of 44% and PMVG alone was associated with a mortality rate of 56%, that rate increased to 72% in patients with both PI and PMVG [8].

Several other CT findings, in addition to PMVG, have been associated with life threatening conditions; these include bowel wall thickening and decreased enhancement, bowel distension, ascites, and mesenteric stranding [12, 13]. In their group of patients, Ko et al. noted that PMVG was only identified in the life-threatening group and never associated with benign PI.

In summary, PI association with PMVG may be due to the mere ability of air to travel up the draining mesenteric veins and therefore could potentially be seen in non-life-threatening cases of PI. Nonetheless, the strong association of combined PI-PMVG with bowel ischemia and infarction, and the increased mortality within that population warrants caution. The identification of PMVG with PI should alert the radiologist to look for accompanying CT features like bowel wall thickening and decreased enhancement, bowel distention, ascites, and mesenteric stranding. In the context of severe clinical presentation and/or these added imaging findings, the presence of PI and PMVG is likely a sign of bowel ischemia. Figure 4 is an illustrative case.

### Portomesenteric vessel occlusion

Mesenteric ischemia due to vascular occlusion may result from arterial inflow obstruction (60–70%) or venous outflow obstruction (5–10%), which differ in pathophysiology, imaging findings, and clinical severity [7, 14].

Arterial occlusion may be caused by arterial emboli or thrombi. Arterial embolic occlusion may be identified by the cut-off sign, which is an abrupt termination of the vessel. Partial occlusion can be identified by the ring sign, which is a central or eccentric luminal defect with a preserved opacified rim [6]. Decreased or cessation of arterial blood flow causes decreased bowel wall enhancement, which can point to an ischemic cause of PI [7, 15]. Necrotic small bowel may appear dilated and filled with fluid or fluid and gas, and bowel walls with a “paper-thin” appearance [7, 16, 18, 19]. Infarcts in other organs such as the kidneys, liver, or spleen will help identify embolic thrombosis. The most common etiology for arterial embolic occlusion is cardiac arrythmia, particularly atrial fibrillation, which presents abruptly and accounts for 50% of cases of arterial occlusion [6, 7, 16].

Another cause of arterial occlusion is arterial thrombosis, which should be suspected in patients with prominent plaques at the origin of the thrombosed vessel in patients with atherosclerotic disease [6, 7]. Atherosclerotic disease-causing thrombosis accounts for 20–30% of cases and may present as acute or chronic due to the gradual progression of the occlusion [7, 16].

Venous thrombosis is identified by rounded hypoattenuating filling defects on portal venous or delayed phases of contrast enhanced CT images and can be visualized in 90% of cases of venous bowel ischemia [18]. Portomesenteric venous thrombosis is often accompanied by bowel mural edema and thickening. Impaired venous outflow increases intramural hydrostatic pressure, and the edematous submucosa will appear hypo-attenuated between the enhancing mucosa and muscularis propria, creating a halo or target appearance [7, 14, 16, 17]. Risk factors for venous thrombosis include inherited or acquired hypercoagulable disorders, malignancy, inflammatory or infectious processes, and venous stasis from immobility [7]. Although venous outflow obstruction produces bowel wall edema and thickening, PI is reported less frequently in isolated venous ischemia compared with acute arterial occlusion, likely reflecting the typically less abrupt compromise in perfusion.

**Fig. 5:**
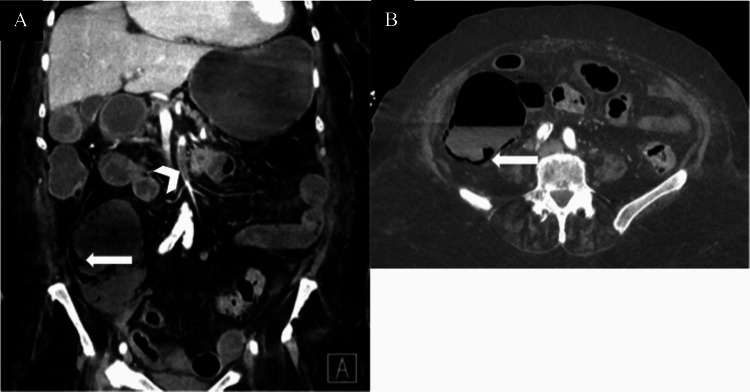
Coronal (**A**) and axial (**B**) CT images show superior mesenteric artery (SMA) occlusion (arrowhead) and PI of the colon (arrows). This patient underwent a SMA thrombectomy and resection of 83 cm of ischemic small bowel and 17 cm of ischemic large bowel including the cecum and appendix

Vascular occlusion has been associated with life-threatening PI [18, 19]. When identified, the possibility of bowel ischemia and necrosis should be considered, especially if pneumatosis is confined to a portion of the small or large bowel in a specific vascular distribution [3, 6, 17]. Figure 5 illustrates a case of bowel ischemia secondary to SMA occlusion.

To summarize, bowel ischemia secondary to vascular disease may be the culprit in settings of PI. Recognizing filling defects in the portomesenteric vessels is essential to make the diagnosis. Additional warning signs that point to ischemia are decreased bowel wall enhancement, bowel wall thinning in arterio-occlusive disease, and bowel wall thickening and halo/target appearance in veno-occlusive disease.

### Fat stranding

Mesenteric fat stranding is an abnormal increase in attenuation of fat caused by increased edema and engorgement of lymphatics from inflammation, injury, or ischemia [20]. It can appear hazy, reticular, or linear [20, 21]. It is a common finding on CT and often directs the radiologist’s attention toward the site of pathology [21]. Results pertaining to the significance of fat stranding in the assessment of PI severity vary among researchers.

Rigiroli et al. reported from their analysis that the three imaging findings that predicted clinically worrisome PI were fat stranding, PMVG, and specific location in the small bowel [19]. Another study evaluating PI in children concluded that both bowel wall thickening and peri-intestinal fat stranding were significant indicators of clinically worrisome causes of PI, like ischemia and inflammation [22]. Copin et al. concluded that mesenteric fat stranding is one of the most sensitive CT findings of acute mesenteric ischemia, up to 96%, though specificity ranges from 28–68% [6].

Other studies evaluating PI and related imaging findings did not find mesenteric fat stranding associated with worrisome PI. Treyaud et al., determined that only PMVG and decreased bowel wall enhancement were indicative of acute intestinal ischemia and that mesenteric fat stranding was not [15]. This study evaluated emergency room patients and etiologies of PI included infection, bowel obstruction, and non-obstructive bowel dilation, etc. which can all present with fat stranding [15].

These seemingly contradicting reports highlight that the presence of fat stranding in the setting of PI is often the indication of an underlying pathological process and rarely innocuous. Mesenteric stranding alone may be associated with infectious or inflammatory conditions like appendicitis, diverticulitis, Crohn’s disease, post-surgical changes, trauma, or malignancy [20, 21]. On the other hand, fat stranding in addition to adjacent decreased bowel enhancement is closely associated with acute mesenteric ischemia. Therefore, although not specific, identifying fat stranding during the assessment of PI may help further narrow the potential causes of illness.

### Ascites

Ascites, or free fluid in the peritoneum, is seen on CT imaging as low-density fluid in dependent spaces and around abdominal organs. It can occur for a multitude of reasons including inflammatory processes, portal hypertension, nephrotic syndrome, neoplasm, etc. [23].

Several studies have concluded that PI with ascites, bowel wall thickening and distension, and decreased bowel wall enhancement point toward an ischemic bowel picture [3, 12, 13, 22]. Dubose et al. looked for predictors of transmural bowel infarction in a large multicentric study that included 500 patients and found a statistically significant correlation between the presence of ascites and pathological PI [18].

A few studies reported no significant association between the presence of peritoneal free fluid and ischemic bowel [15, 19]. A possible explanation could be that in patients with other comorbidities, ascites may be secondary to pathologies preceding the development of PI. In the absence of other explanations like significant cardiac, liver, or renal disease, the presence of ascites in the setting of PI is often regarded as an ominous sign.

### Pneumoperitoneum

**Fig. 6:**
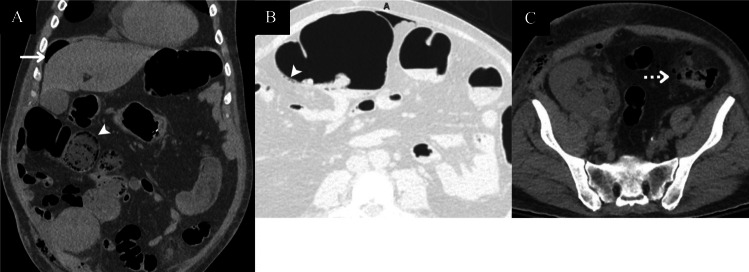
52-year-old patient with acute perforated sigmoid diverticulitis. Coronal CT image **A** shows PI of the cecum (arrowhead) and pneumoperitoneum (solid arrow). Axial CT image **B** shows PI of the cecum (arrowhead). Axial CT image **C** shows perforated sigmoid diverticulitis (dashed arrow). This patient underwent an exploratory laparotomy which showed a dilated cecum with no signs of ischemia

Pneumoperitoneum, or free air in the peritoneal cavity, is usually seen in non-dependent areas of the abdomen. It can be caused by perforation of a hollow viscus (Fig. 6), iatrogenic causes including abdominal surgery or endoscopy, infections, etc. The most worrisome cause of pneumoperitoneum associated with PI is the progression of bowel ischemia leading to perforation, which requires emergent surgical intervention.

**Fig. 7:**
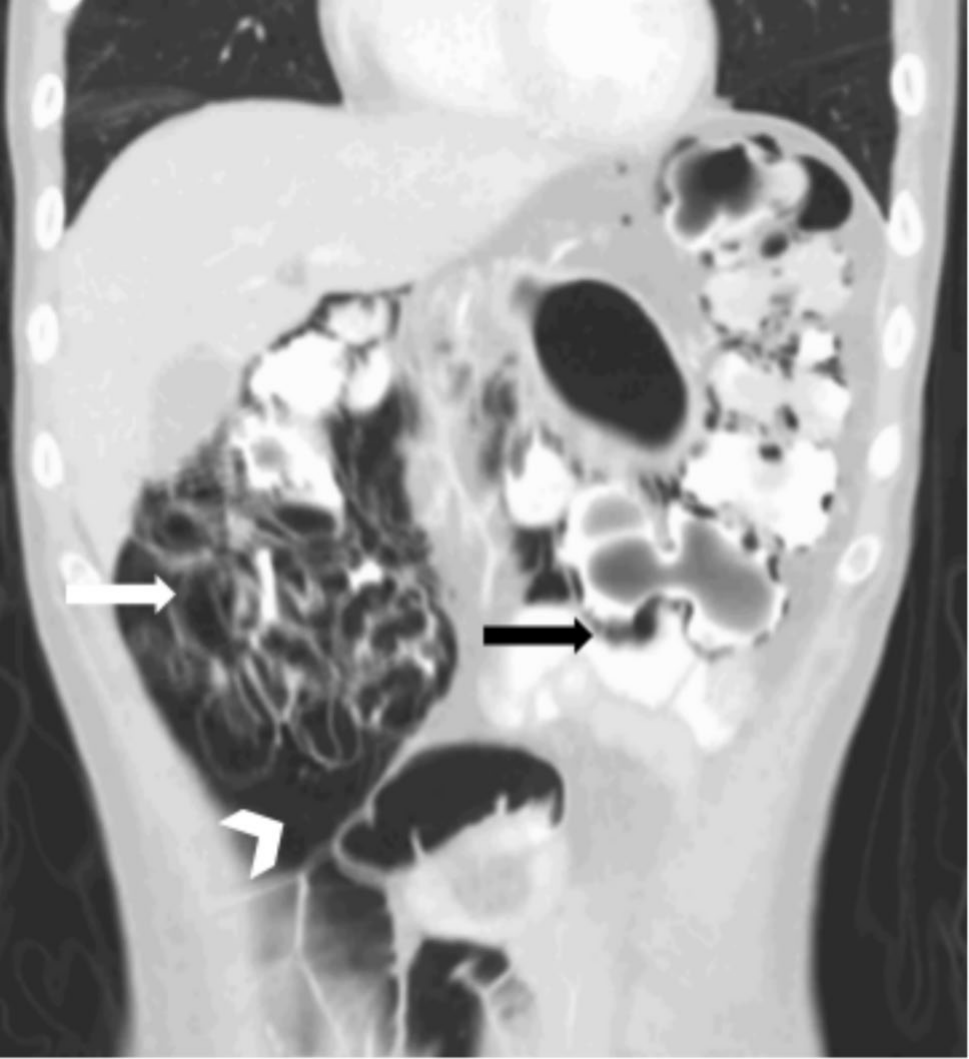
34-year-old male with HIV/AIDS on high dose steroids. Coronal CT image shows PI of the colon (arrows) and adjacent pneumoperitoneum (arrowhead). This patient was asymptomatic and treated with conservative management including bowel rest and intravenous antibiotics

**Fig. 8:**
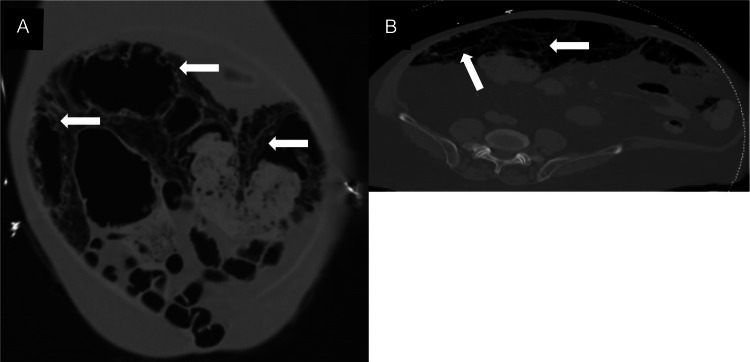
54-year-old with history of Crohn’s disease and disseminated Mycobacterium Avium Complex (MAC) on long-term steroids, presents with back and abdominal pain with nausea. Coronal (**A**) and axial (**B**) CT images show PI of the colon with cystic and linear patterns (arrows) and extensive air throughout the mesentery. Workup revealed mild tenderness to palpation, normal WBC and lactate levels. The patient was treated conservatively with bowel rest, IV fluids, and antibiotics

**Fig. 9:**
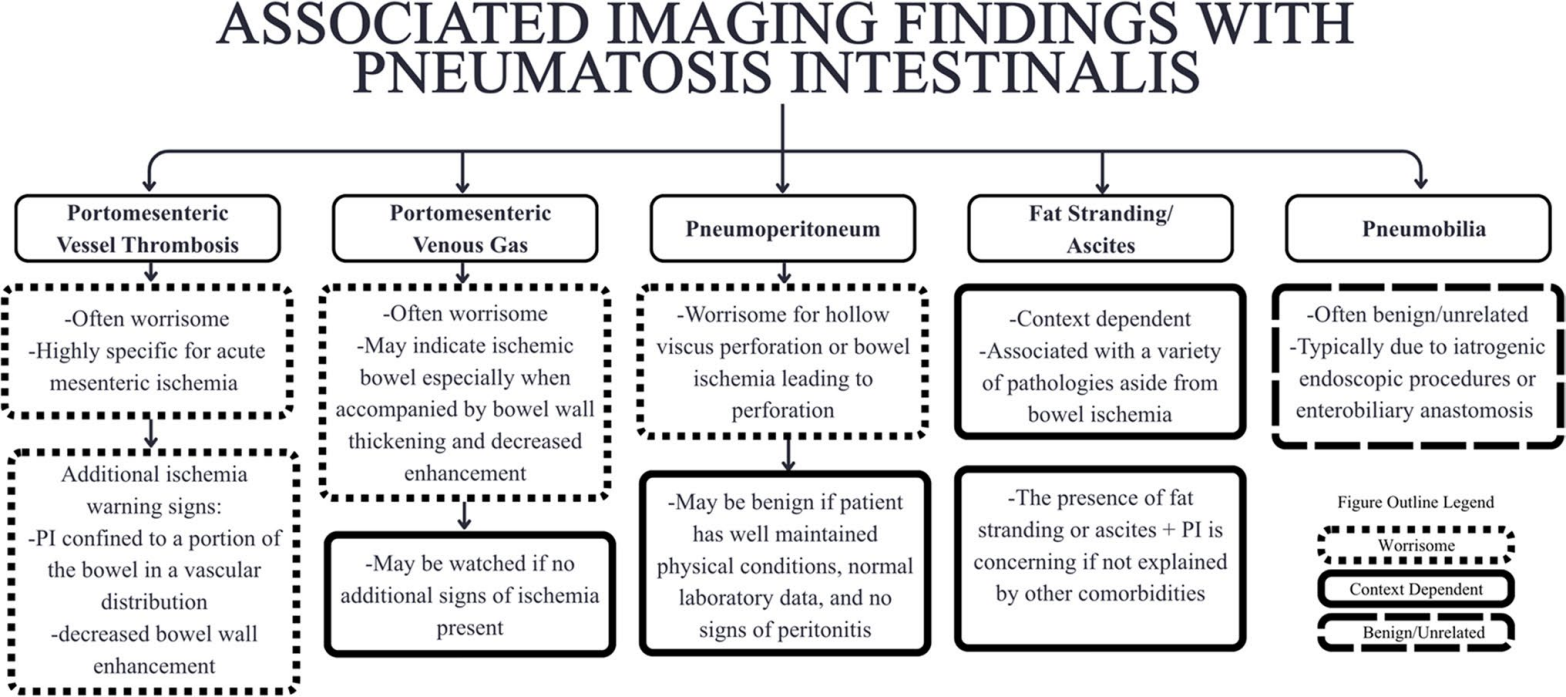
Summary chart of imaging findings associated with pneumatosis intestinalis (PI) and their clinical implications

However, under certain conditions, especially if the patient has well-maintained physical conditions, normal laboratory data, and no signs of peritonitis, pneumoperitoneum alone with PI may not be worrisome [24, 25]. For example, Adachi et el., discovered 20/21 cases of PI with pneumoperitoneum were benign with no evidence of bowel wall discontinuity, bowel-wall thickening, fat stranding, or abscesses [24]. In cases of primary PI also known as Pneumatosis Cystoides Intestinalis, rupture of the intramural filled cysts can result in pneumoperitoneum [24]. In summary, the presence of pneumoperitoneum with PI should raise immediate concern due to the inherent severity of hollow organ perforation. In cases of primary PI or in the absence of concomitant findings to suggest bowel ischemia and/or perforation, a more conservative approach can be considered. Figures 7 and 8 are illustrative cases of PI associated with pneumoperitoneum that benefited from non-operative management. Figure 9 is a summary chart of imaging findings associated with PI.

## Nuances in clinical interpretation

Imaging characteristics are only part of the diagnostic picture, and understanding their implications requires examining the broader challenges involved in distinguishing benign from pathologic PI. In this section, we discuss clinical markers involved in interpreting the severity of PI and the nuances regarding certain populations.

### Laboratory markers

Elevated lactate levels have historically been regarded as an important clinical marker in evaluating the severity of PI and have often been interpreted as suggestive of pathologic disease. Several studies have supported this association, identifying increased lactate as a predictor of pathologic PI [18, 26–28]. The proposed mechanism relates to tissue hypoxia from impaired mesenteric perfusion: when intestinal blood flow is compromised, ischemic bowel shifts toward anaerobic metabolism, resulting in lactate production that exceeds hepatic clearance.

More recent investigations, however, have challenged the reliability of lactate as a consistent predictor of pathologic PI. While earlier studies emphasized its diagnostic value, recent analyses did not demonstrate a significant association between elevated lactate levels and pathologic PI [19, 29]. This discrepancy likely reflects the nonspecific nature of elevated lactate, which typically rises only after significant or irreversible bowel injury has occurred [30]. While elevated lactate can be caused by tissue hypoxia from impaired mesenteric perfusion, it is not specific to ischemia. It may also arise from aerobic glycolysis triggered by stress-related catecholamine surges, particularly in critically ill patients with sepsis or shock who may develop PI through non-ischemic mechanisms [31]. The inconsistency in elevated lactate as a predictor of pathologic PI underscores the diverse mechanisms that can produce PI.

Leukocytosis has similarly been considered a marker of pathologic PI and underlying physiological stress. Multiple studies have reported an association between elevated white blood cell (WBC) count and pathologic PI [15, 19, 27, 29], supporting its potential role as an indicator of bowel ischemia or necrosis. However, the strength of this association varies across studies. Greenstein et al. demonstrated significance on multivariable analysis, whereas several others found significance only on univariable analysis [18, 26, 27, 32]. In contrast, Duron et al. reported no significant association on either analysis [33].

As with lactate, the limited specificity of leukocytosis likely contributes to these conflicting findings. WBC count may be elevated in non-ischemic PI due to infection, mucosal injury from inflammatory bowel disease, or immunosuppression, among other causes [4, 19, 29, 34].

These observations suggest that while elevated lactate and leukocytosis may raise concern for pathologic PI, neither laboratory marker is sufficiently specific nor sensitive when considered in isolation [30, 35]. Accurate risk stratification requires integration of laboratory findings with imaging features, physical examination, and the broader clinical context.

### Considerations in special populations

#### Lung disease, mechanical ventilation, motility and autoimmune disorders

The occurrence of PI in certain thoracic conditions is well documented. In patients with emphysema, chronic obstructive pulmonary disease and cystic fibrosis, it is hypothesized that air from alveolar rupture can dissect along the vasculature and reach the bowel wall [2–4, 12, 34]. These cases are often benign and follow a benign course [3]. Similarly, increased alveolar pressure during mechanical ventilation may result in barotrauma leading to escaping of air from the alveoli [34].

Disorders affecting intestinal motility, for example chronic constipation and neurological disorders like cerebral palsy, may be associated with PI through increased intraluminal pressure forcing air within the walls.

Several autoimmune diseases have also been linked to the development of PI. Scleroderma, SLE and myopathies such as dermatomyositis are notable associations [36, 37]. A combination of factors including mucosal damage from intestinal vasculitis, bacterial overgrowth and immune-related therapy might be at play. As expected in such a diverse group of disorders, clinical presentation varies, and the significance of PI is derived from the overall patients’ condition.

#### Corticosteroids users, transplants recipients and oncology patients

The use of certain medications has long been known to be a possible cause of PI, with corticosteroids often cited as the most common culprit [3, 38]. The advent of targeted and immunotherapy in the last few decades and their increasing use in solid and hematologic tumors has also come with increased recognition of adverse effects including the development of PI. It is theorized that penetration of air in the bowel wall may be secondary to tissue damage and/or from bowel inflammation secondary to activation of the immune system that leads to breaks in the bowel wall and intestinal inflammation [39, 40]. Common offenders, according to a systematic review of 62 articles conducted in 2022, are cytotoxic and targeted therapies, specifically kinases inhibitors and monoclonal antibodies [41]. Several other reports and studies have brought attention to the occurrence of PI specifically in patients receiving immune check point inhibitors [39, 42, 43]. A large analysis of the FDA Adverse Event Reporting System spanning 17 years found over a thousand cases of PI related to administration of steroids or immune checkpoint inhibitors [44]. Likewise, the occurrence of PI in solid organs transplants and hematopoietic cell transplants recipients has been linked to the use of steroids and other immunosuppressant agents in several reports [45–48]. Similar to PI from other causes, severity of disease and outcomes vary. In some cases, PI is an incidental finding in a routine or unrelated scan, that resolves spontaneously or improves with conservative management; whereas other cases can present with symptoms as severe as an acute abdomen and develop complications including perforation, fluid collections and even death [39, 49–52].

## Management

The approach to PI management relies on radiologic findings, symptomatology, findings from physical examination, and laboratory data. Depending on these factors, a patient may benefit from non-operative management or undergo surgical intervention, addressing both the PI and the underlying cause. The initial step in management is always to determine if urgent surgery is needed [53].

### Surgical management

Currently there are no established guidelines for the management of PI; data are derived from case series and retrospective cohort studies, and management may vary among institutions. Two universal pillars guiding the decision for surgical interventions are the probability of bowel compromise and a critically ill and/or unstable patient.

According to various studies, the highest predictive risk factors for poor prognosis related to PI include hypotension, peritonitis, a lactate level greater than 2 mmol/L, acute renal failure, and mechanical ventilation [18]. Other studies indicate that higher Acute Physiology and Chronic Health Evaluation (APACHE) II scores, presence of portal venous gas on imaging, acute rise in creatinine, bowel dilatation, age > 60, among others also help guide decisions regarding surgical management for the appropriate patient [53–58].

Surgical intervention usually consists of an exploratory laparotomy which may lead to resection of ischemic or compromised bowel, intestinal diversion with creation of ostomy. If no compromised bowel is identified, surgeons may abort the procedure and close incision. Alternatively, they may opt for a temporary closure with plans for a second look in 24–48 h to reevaluate evolving pathology [54, 59, 60].

In addition, cases of bowel ischemia with PMVT as an underlying cause can be treated with prompt revascularization through open or catheter-directed thrombectomy/thrombolytic procedures and systemic anticoagulation [4, 6, 14, 17].

### Endoscopy

Endoscopy can accurately make the diagnosis of pneumatosis cystoides intestinalis (PCI) or primary PI through direct visualization of gas-filled cysts within the bowel wall [61].

In rare instances where intestinal obstruction is caused by extensive PCI, puncture of the cysts during endoscopy can simultaneously confirm and relieve the obstruction. Sclerotherapy can also be performed during endoscopy to prevent cyst re-expansion [4, 62, 63].

### Non-operative management

Non-operative/medical management is initiated as soon as the need for urgent surgical intervention is ruled out. It aims to address various aspects of PI physiopathology, provides support to minimize disease progression and foster recovery. Therapy options include antibiotics, bowel rest, and oxygen therapy. In select clinical scenarios, particularly when PI is identified incidentally in an asymptomatic patient with no history of disease process or medication use related to PI, observation alone may be appropriate [64].

Antibiotics are the mainstay treatment for non-surgical patients with PI. A common explanation for the presence of PI is the theory that gas-producing or other types of bacteria may invade the bowel wall directly or alter intraluminal gas, causing accumulation of gas within the wall due to increased pressure, mucosal injury, or both. Antimicrobial therapy is therefore used to eliminate gas from that origin [13, 65, 66].

Hyperbaric oxygen therapy reduces symptoms by lowering gas pressure in veins and acts as a toxin for anaerobic bacteria in the intestinal tract [66, 67]. Therapy can be enhanced with humidified oxygen via a mask or nasal cannula (4 to 6 L/min). Oxygen should be used for two days after cyst disappearance to prevent recurrence [62, 66].

Additionally, several case series and reports have demonstrated the association between prolonged steroid and chemotherapy use with the presence of PI and therefore decrement or discontinuation of immunosuppressive medications may promote remission of PI [44, 47, 49, 51, 66, 68, 69].

## Risk stratification and future perspectives

There are no current established guidelines for predicting the probability of pathologic or benign PI. Pathologic PI refers to clinically significant intestinal injury, such as ischemia, necrosis, or perforation, and may require urgent intervention. Benign PI, often seen in pulmonary or other non-ischemic conditions, is usually asymptomatic with normal labs and can be managed conservatively [3]. Distinguishing between the two is essential for guiding appropriate monitoring and treatment. Several studies have proposed decision-making algorithms for the evaluation and management of PI. For example, DuBose et al. developed a pathway that prioritized elevated lactate level (≥ 2.0 mmol/L), followed by assessment for shock or hypotension and peritonitis [18]. A study by the American Association for the Surgery of Trauma evaluated several clinical prediction models, and derived a model that incorporated hemoglobin level, lactate ≥ 2 mmol/L, INR, small-bowel involvement, acute kidney injury, ascites, and clinical signs of peritonitis [26].

Other studies expanded their frameworks to include radiological evaluations. Rieser et al. developed a risk scoring system to identify individuals with high likelihood of intestinal ischemia. This study identified five factors associated with pathologic PI: small bowel PI, age 70 years or older, heart rate 110 bpm or greater, lactate ≥ 2 mmol/L, and neutrophil–lymphocyte ratio 10 or greater [27].

More recently, Rigiroli et al. established a combined model incorporating both imaging and clinical variables to distinguish patients with pathologic PI. They included mesenteric or portal venous gas, perilesional fat stranding, patient age, white blood cell (WBC) count, creatinine level, bowel distention, abdominal guarding or rebound tenderness, and hemodynamic instability [19]. Similarly, Song et al. evaluated predictors of pathologic PI and created a nomogram to calculate the likelihood that a patient with PI would benefit from surgical intervention. The predictors they found included presence of peritonitis, portovenous gas, multisegments PI, leukocytosis, vasopressor requirement, and end organ injury [29].

Mather and Diaz recently proposed a grading system of PI which incorporates history, physical examination, laboratory values, and imaging findings. An accompanying decision tree outlines corresponding management most appropriate for the degree of severity [64]. A clear definition for each grade allows uniformity of language among healthcare providers, better prognosis assessment and enable more targeted interventions (Table 1). These publications highlight the growing effort to codify risk stratification and guide management of the diverse population of patients with PI.

**Table 1:**
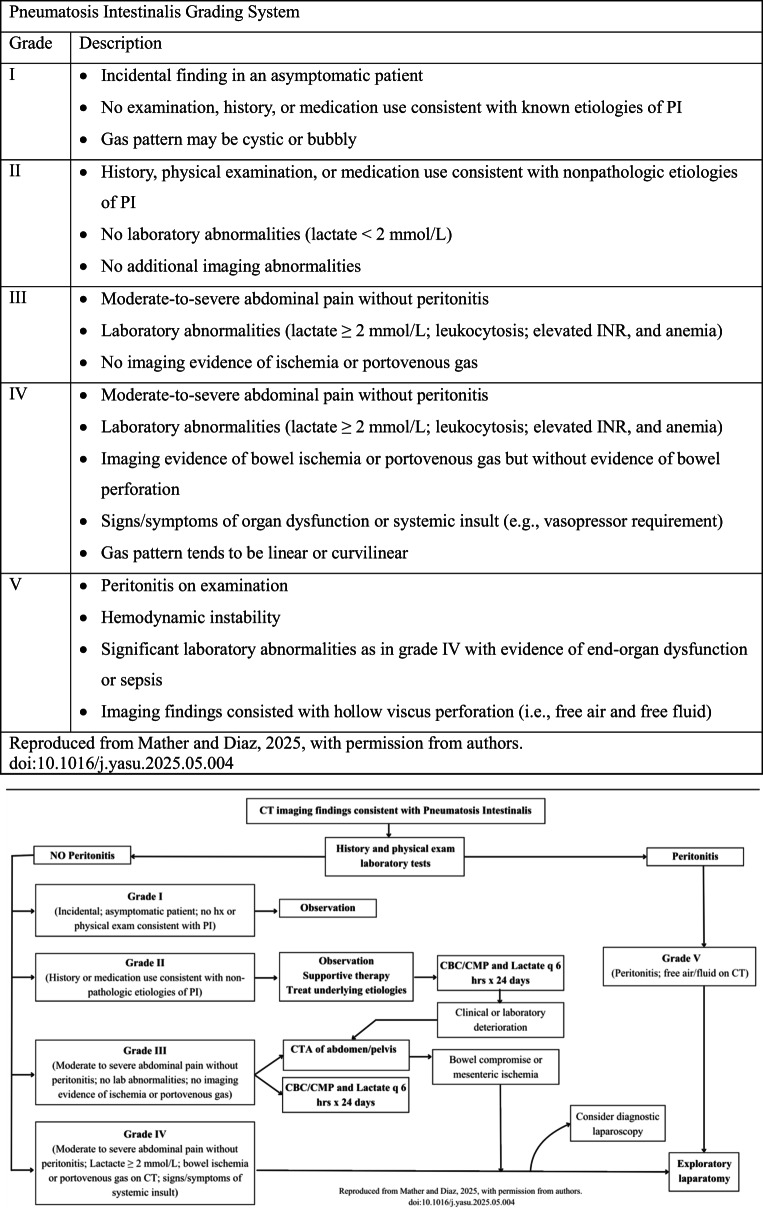
Pneumatosis intestinalis grading system and corresponding management flow chart. Reproduced with permission from authors [64]

Over the years, the gap in knowledge has certainly been reduced and resulted in a better comprehension of the numerous possible scenarios, conditions, and medication use associated with PI. Unfortunately, there remain gray areas in knowledge and controversies in either interpretation of findings or approach to treatment.

Mechanical ventilation is commonly cited to illustrate the mechanical theory in the development of PI from relatively benign etiologies. Conversely, it is associated with a higher risk of clinically significant ischemia in some cohorts. Whether the bad prognosis is due to the underlying pathology that requires ventilation or caused by ischemia is not definitely elucidated. Moreover, in certain circumstances it might be difficult to determine if PI is simply a complication of ventilation, or a sign that bowel ischemia might be developing. While it is difficult to separate correlation from causation, it is possible that more than one contributing factor exists.

There is strong evidence to suggest that steroids and other immunotherapies are linked to the development of PI, and many report that discontinuity of these agents in these cases improves outcome [70]. Steroids are also recommended therapy options to treat various immune-related adverse events in patients treated with immune checkpoint inhibitors [71].

While a case-based approach might be the most sensible approach, more research is needed to further elucidate physiopathology of causal links and therapeutic effects. In the meantime, concerted efforts should be undertaken to unify proposed algorithms and establish clear and comprehensive guidelines.

## Conclusion

PI has been extensively studied, yet it continues to present a diagnostic and management challenge due to its wide spectrum of underlying etiologies and overlapping imaging and clinical features. Its clinical significance ranges from benign, self-limited processes to life-threatening bowel ischemia. Imaging features such as concomitant PMVG, thrombosis of the portomesenteric vessels, thinning or thickening of the bowel wall, and abnormal bowel enhancement should raise major concern for bowel ischemia. The significance of pneumoperitoneum in the setting of PI is context-dependent; while it is often associated with bowel necrosis and perforation, a case-based approach is prudent in immunosuppressed patients, and in the absence of worrisome clinical signs and data, to avoid excessive surgical intervention.

We also discussed laboratory markers historically used to indicate poor prognosis, including elevated lactate and elevated WBC count. Inconsistencies among studies on the predictive value of these clinical markers likely reflect differences in patient populations, study size, and methodology, and underscore the variable presentations of PI. These variations highlight the necessity of interpreting laboratory findings within a comprehensive clinical and imaging-based risk stratification framework.

In this review, we also addressed nuances in management of patients receiving chemotherapy or immunosuppressive therapy, and summarized emerging frameworks for risk stratification, including grading systems and integrated diagnostic algorithms.

Moving forward, the field would benefit from a consensus-driven, standardized risk stratification algorithm that integrates imaging findings, laboratory data, and clinical presentation across diverse patient populations. Such uniform guidelines would improve diagnostic accuracy, optimize management strategies, and help distinguish between benign and pathologic PI in a consistent and reproducible manner.

## Data Availability

This review article did not generate or analyze any new data.
